# The association between residential area characteristics and mental health outcomes among men and women in Belgium

**DOI:** 10.1186/0778-7367-69-3

**Published:** 2011-10-24

**Authors:** Elise Pattyn, Lore Van Praag, Mieke Verhaeghe, Katia Levecque, Piet Bracke

**Affiliations:** 1 HeDeRa (Health & Demographic Research), Department of Sociology, Ghent University, Korte Meer 5, 9000 Ghent, Belgium; 2 CuDOS (Cultural Diversity: Opportunities and Socialization), Department of Sociology, Ghent University, Korte Meer 5, 9000 Ghent, Belgium; 3 Research Foundation (FWO), Flanders, Belgium

## Abstract

**Aim:**

Recently, interest has grown in the association between contextual factors and health outcomes. This study questions whether mental health complaints vary according to the socio-economic characteristics of the residential area where people live. The gender-specific patterns are studied.

**Methods:**

Complaints of depression and generalized anxiety were measured by means of the relevant subscales of the Symptoms Checklist 90-Revised. Multilevel models were estimated with PASW statistics 18, based on a unique dataset, constructed by merging data from the Belgian Health Interview Surveys from 2001 and 2004 with data from 264 municipalities derived from Statistics Belgium and the General Socio-Economic Survey.

**Main findings:**

The results of this exploratory study indicate that the local unemployment rate is associated with complaints of depression among women.

**Conclusion:**

This study suggests that policy should approach the male and female population differently when implementing mental health prevention campaigns.

## Introduction

The mental health epidemic contributes extensively to the global burden of disease. In middle- and high-income countries, depression is the primary cause of loss of health [1]. Due to the high prevalence of mental disorders, it is crucial to examine which factors determine mental health problems. Current research in Belgium concerning the determinants of mental wellbeing primarily draws attention to individual determinants. However, international empirical research has demonstrated that health outcomes differ according to the residential area in which a person lives. Studies in Great Britain [2-7], The Netherlands [8], Canada [9], Sweden [10] and the United States [11,12] showed that these differences can partly be attributed to contextual effects. This study will fill this research gap by studying the association between contextual factors and mental health complaints among the general Belgian population. To the best of our knowledge, only Lorant and colleagues [13] studied contextual influences on mental health in Belgium, but this among a selective population of migrants.

A diverse set of socio-economic characteristics of the residential area has been suggested as affecting the mental health of its inhabitants [4,11,14-17]. The social conditions of an individual's neighborhood operate beyond the impact of one's personal socio-economic status [18-20]. The socioeconomic climate of a residential area will be addressed by referring to the local unemployment rate and the median area income. The local unemployment rate can be seen as a source of stress, since it reflects the present and future work conditions of the inhabitants [4,21-23]. The median area income is related to mental health complaints in the sense that a higher income has a negative impact on the amount of complaints reported, as stated by several authors. Moreover, by taking the median area income into account, other aspects of the local unemployment rate, beside the level of income deprivation, will be looked upon [15,24-26].

Different models highlight the impact of residential area characteristics on various types of mental health outcomes such as the structural characteristics model, the neighborhood disorder model, the environmental stress model [27] and the social stress model [28]. The research questions of this study are derived from the latter model, which has been supported by empirical research of Ross [29] and Aneshensel and Sucoff [30]. When trying to explain the variation in mental health outcomes, the social stress model posits that stressful chronic life difficulties cause psychological stress and that psychological stress contributes to mental health problems. The model identifies three components of the stress process: stressors, moderators and outcomes. First, Pearlin [28] argues that the social environment to which all inhabitants of a residential area are exposed, contains a set of potential chronic stressors he calls 'ambient strains' which can affect mental health in a detrimental way. Turner, Wheaton and Lloyd [31] stress that chronic stressors, such as living under conditions of economic hardship, contribute more to social group differences in mental health than do discrete life events. Second, the outcomes refer to psychological distress. Following Kawachi and Berkman [32], this study will highlight two types of internalizing disorders, namely complaints of depression and generalized anxiety, which Dohrenwend et al. [33] define as non-specific psychological distress. Third, moderators interfere with the association between stressors and distress. Moderators are resources that people mobilize to regulate the effects of the stressors [34]. Social support is a well known buffer of stress; it influences the way people perceive stress and may help prevent psychological distress [35,36].

With regard to the aim of this study, the social stress model expresses two interesting hypotheses. A first hypothesis is that the variation in mental health outcomes can be explained by differences in vulnerability to environmental stressors, as people assign specific positive and negative values to potential stressors [37,38]. A second hypothesis is that the variation can be attributed to differences in access to adequate supportive relationships that help individuals to cope with stress [32,38]. Moreover, the value people assign to stressors and the resources they possess vary among social groups. This study will highlight gender differences, as several studies revealed that the residential area affects women's health more profoundly, compared to men [39-41]. However, the empirical underpinnings of this finding are inconsistent [42-50]. Referring to the two formulated hypotheses, two nuances can be made with regard to gender differences. First, concerning vulnerability, it can be suggested that it is easier for women to cope with job loss and loss of income, due to the existence of alternative family roles following the role enhancement theory [51]. Second, concerning the buffering effect of social support, Belle [52] suggests that women mobilize more social support during periods of stress than men. Men merely seek support from their spouse, whereas women are much more likely to rely on their child, a close relative, or a friend as their confidant.

Previous research outlined a series of risk factors of mental health complaints that cannot be left aside in the analyses. At the individual level, the socio-economic status of the individual should be taken into account. Unemployment at the individual level has a detrimental effect on mental health too, as employment can be considered as a source of self-sufficiency, recognition and social contact [4,23]. The relationship between education and mental wellbeing is another consistent finding in the literature [53]. Next to this, poverty constitutes a serious threat to mental wellbeing [13,54]. Furthermore, some international studies showed a curvilinear relationship in which the highest prevalence of complaints of depression was found among adolescents and seniors [55,56]. With regard to anxiety disorders, particularly people younger than 30 years old were confronted with complaints of generalized anxiety [57]. In addition, the type of household seems to have an impact. People with a partner, married or cohabiting, generally report lower levels of mental distress [58]. Inconsistent findings were found on whether or not having children is beneficial or detrimental [51]. Last but not least, at the residential area level, the population density should be controlled for. The population density is the amount of people who live in the same area and can be considered as an indicator of urbanization [59,60]. But it can also serve as a stressor which is related to social pathology according to Taylor et al. [61] and Greiner et al. [62]. Yet another study of Weich et al. [63] found mixed results.

In sum, this exploratory study will study the association between socio-economic characteristics of the residential area and complaints of depression and generalized anxiety among the general Belgian population. The research question is whether the amount of complaints of depression and generalized anxiety differs according to the local unemployment rate and the median area income, net of individual characteristics. More specifically, we focus on gender-specific patterns. This study questions whether women's mental health is more affected by characteristics of the residential area. Therefore, a conservative test is applied. Does the association between residential area characteristics and women's mental health hold when on the one hand, socio-economic characteristics of the residential area are focus of attention and on the other, the availability of social support is taken into account? We hypothesize that women will be less vulnerable to socio-economic stressors and that women get more stress relief thanks to their reliance on social support, compared to their male counterparts.

## Methods

### Sample

The individual level variables are obtained from the Health Interview Survey (HIS). The aim of the HIS is to gather information about the physical and mental health status and the use of preventive and curative health services. This survey will allow researchers to detect demographical, socio-economic and cultural gradients in different aspects of health. Data gathering was organized by the Scientific Institute of Public Health, based on a multistage stratified cluster sample in which selected households were geographically clustered according to their municipality code. For part of the questions, e.g., those concerning mental health, proxies were not allowed. The provided cross-sectional data are representative of the Belgian population older than 15 years old within collective and private households with an oversampling of the elderly in the HIS of 2004. The sample does not include institutionalized individuals (e.g. in psychiatric care or in prison), with the exception of the elderly living in nursing homes. Household characteristics were assessed by means of a written questionnaire, filled in by one person of the household. Within each household, a maximum of three other individuals were questioned too. Individual characteristics were assessed by means of a verbal and/or written questionnaire administered to the selected household members [64,65].

In this study, data from two waves, carried out during the calendar year of 2001 (N = 12.111) and 2004 (N = 12.945), of the Belgian National Household Interview Survey are pooled to enlarge the number of cases. The response rate at the household level was 61.4% for both samples. After deleting cases that missed substantive information, the research sample regarding complaints of depression includes 18,174 respondents in total.

When we take complaints of generalized anxiety into consideration, the total sample consists of 18,109 respondents. This represents a male population of 47.3% and a female population of 52.7%. The data at the individual level were merged with residential area-level data obtained from Statistics Belgium [66] and the General Socio-economic survey of 2001, based on corresponding municipality codes. The latter survey was obligatory for all inhabitants of Belgium and replaced the formerly conducted census. 20% of the data could be obtained for the present study. In total, 264 municipalities are included, which constitutes 44.8% of the total number of municipalities in Belgium (589) with a minimum number of inhabitants of 2,219 and a maximum of 447,139.

### Dependent variables

The mental health outcomes were measured by means of the relevant subscales of the Symptoms Checklist-90-Revised. This is a self-report scale developed by Derogatis [67] which considers different dimensions of psychopathology. The scale has been used in both general population surveys as well as in patient populations. This study discusses *complaints of depression and generalized anxiety*. Depressive complaints refer to feeling down, a low level of energy, feelings of guilt, low self-esteem, suicidal thoughts, loneliness and worrying. Complaints of generalized anxiety include heart palpitations, feeling tensed, being restless, sudden fears, trembling, being scared and panicking. The subscale scores of complaints of depression and generalized anxiety are constructed by the mean value of all valid items. The subscales assess the feelings of the respondent during the past week by means of answering categories that range from 'never'(1), 'seldom'(2), 'sometimes'(3), 'a little'(4) to 'always'(5) [68]. Missing values were substituted, for a maximum of three items, by the most correlated item in the scale [69], resulting in an item response rate of 91.1%. The scales have been extensively used in international literature and have been shown to have excellent reliability and validity in the general Belgian population according to a study of Levecque [68]. First, the subscale of complaints of depression has a Cronbach's Alpha of 0.90 and the generalized anxiety subscale has a Cronbach's Alpha of 0.88. Nunally [70] considers the internal validity as satisfying when the Cronbach's Alpha is higher than 0.80. Second, the reliability is expressed by means of the internal consistency of the scale, namely the correlation between the different items. The inter-item correlation varies between 0.45 and 0.79 with a mean value of 0.64 for complaints of depression. In case of complaints of generalized anxiety, it fluctuates between 0.30 and 0.66 with a mean value of 0.44. So the separate symptoms are highly coherent. Moreover, the confirmatory factor analyses confirm that the scales adequately measure one-dimensional latent constructs of depression and generalized anxiety. The model fit indicator, Comparative Fit Index, should approximate 0.90 to be acceptable according to Hoyle & Panter [71]. This amounts to 0.93 for the scale of depressive complaints and to 0.89 for the scale of generalized anxiety.

### Individual characteristics

The key variables at the individual level are gender and social support. *Gender *is represented as a dummy variable: men receive the score of 1 and women are defined as the reference category. *Social support *is assessed using the Medical Outcomes Study (MOS) Social Support scale, as composed by Sherbourne and Stewart [72]. This scale questions the function and quality of social contacts. It refers to the perceived affective, emotional and instrumental support one receives and to the positive social interaction one gets. These 19 Likert-type items range from 'never'(1), 'rarely'(2), 'sometimes'(3), 'often'(4) to 'always'(5). If more than 4 items were missing, the whole scale was set as missing and excluded from the analyses. Otherwise, the mean score of the valid items was calculated.

Several control variables are included in the model. The socio-economic status of the respondent is expressed by means of its current employment status, educational level and household income. The *current employment status *is expressed by means of the following categories: 'retired', 'disabled', 'unemployed', 'student and others' and 'househusband/wife', which are compared to the reference category 'in paid labor'. The *educational level *is expressed by means of the highest diploma or degree one has attained and is subdivided into four categories: 'no diploma or primary education', 'lower secondary', 'higher secondary' and the reference category 'tertiary education'. The *equivalent household income *is based on the modified OECD scale (Organization for Economic Co-operation and Development) [73] which gives weight to the household income according to the composition of the household. The first adult of the household is given a weight of 1, the other adults are given a weight of 0.5 and each child under 18 years is given a weight of 0.3. These weighted household incomes were subdivided in six categories: 'less than 750 euro', '750-1000 euro', '1000-1500 euro', '1500-2500 euro', 'income missing' and the reference category 'more than 2500 euro'. In addition, age and household type are controlled for. *Age *is measured in years. *Household type *refers to one of the following: 'single', 'single with child(ren)', 'couple' (partners living together), 'complex household' (co-habitants, but no partners) and 'couple with children' (reference category).

### Residential area characteristics

The two variables that refer to the socio-economic climate of the residential area are local unemployment rate and median household income. The *local unemployment rate *is constructed by the ratio of the unemployed population (whether or not currently seeking a job), compared to the sum of all individuals aged 15-64 years old, or the latent labor force. This measure is based on the data of the 2001 census, General Socio-economic Survey. The *median household income (in euros) *is based on the distribution of the yearly tax declarations of the inhabitants of the residential area. As control variable, the *population density *is measured by means of the number of inhabitants per square kilometre. The latter two indicators are based on data of Statistics Belgium (2004).

### Analyses

First of all, the intraclass correlation coefficient (ICC) was calculated to estimate the proportion of variance of mental health complaints that could be explained by residential area characteristics, making use of the intercept-only model of the Mixed Models procedure in PASW 18 Statistics. The between-group variance was divided by the total variance of the mental health complaints [74]. Subsequently, by means of multilevel modeling, namely the Restricted Maximum Likelihood estimation method, we explored the association between residential area characteristics and complaints of depression and generalized anxiety, net of individual characteristics. The multilevel models were conducted for men and women separately to check for gender-specific patterns. No weights to control for the design of the sample were applied on the individual level, since the data were clustered according to their residential area and were analyzed at the municipality level.

Before conducting the analyses, several variables were transformed. The mental health outcomes have a skewed distribution. To account for this distribution, the dependent variables were logarithmically transformed [75,76]. Furthermore, they were centered around their overall mean. Next to this, some individual characteristics were centered around the overall mean. This was the case for age and social support. Moreover, the original values of age were divided by ten to enlarge the parameter estimates. In addition, all the residential area characteristics were centered around the overall mean and in the case of median household income and population density, their original values were divided by one million to enlarge the parameter estimates.

## Results

The descriptive statistics are presented in Table 1.

**Table 1:** Sample descriptives, Belgium 2001-2004

Mental health outcomes (before log transformation)#				
	**MEN**		**WOMEN**	
	***Mean*** ***(Min. - Max.)***	***S.D.***	***Mean*** ***(Min. - Max.)***	***S.D.***

Complaints of depression	1.3 (1 - 5)	0.5	1.4 (1 - 5)	0.6
Complaints of generalised anxiety	1.2 (1 - 5)	0.4	1.3 ( 1 - 5)	0.5

**Independent variables at the residential area level**				

	**Median (Min. - Max.)**			

Local unemployment rate*	0.11 (0.03- 0.34)			
Median area income^§ ^(in euros)	18,878 (13,379 - 24,352)			
Population density^§^	599 (21 -19,480)			

**Independent variables at the individual level**				

	**MEN**		**WOMEN**	

	***Mean*** ***(Min. - Max.)***	***S.D.***	***Mean*** ***(Min. - Max.)***	***S.D.***

Social support	4.0 (1 - 5)	1.0	4.0 (1 - 5)	1.0
Age	47.5 (15 - 98)	18.9	48.9 (15 - 101)	19.8

	***%***		***%***	

**Current employment situation**^**#**^				
*Retired*	26.4		26	
*Disabled*	2.6		2.5	
*Unemployed*	4.8		6.1	
*Student and others*	0.3		12.6	
*Househusband/wife*	1.4		2.4	
*Paid job (ref.)*				
**Educational level**^**#**^				
*No diploma or primary education*	15		19.2	
*Lower secondary*	18.4		19.2	
*Higher secondary*	28.3		24.3	
*Tertiary education (ref.)*				
**Equivalent income of the household**^**#**^				
*< 750 €*	4.5		6.1	
*750 - 1.000 €*	7.9		11.2	
*1.000 - 1.500 €*	20.4		20.2	
*1.500 - 2.500 €*	30.1		28.2	
*> 2500 € (ref.)*	13.0		13.4	
**Household type**^**#**^				
*Single*	19.7		23.9	
*Single with child(ren)*	2.3		6.8	
*Couple*	34.4		29.7	
*Complex household*	14.5		13.0	
*Couple with child(ren) (ref.)*				

ref.: reference category in multilevel regression models

*: General Socio-economic Survey, 2001

§: Statistics Belgium, 2004

#: Health Interview Survey, 2001 & 2004

Second, the results of the *multilevel regression analyses *are presented in Table 2 and 3. As the local unemployment rate and the median area income are correlated (*Pearson Correlation: -0.767, significant at the .01-level)*, we first applied step by step modeling. Yet these results did not differ from the results of the final model, in which all residential area characteristics were added. As a consequence, only the results of the final model are tabulated.

**Table 2:** Multilevel regression analyses: association between residential area characteristics and complaints of depression among men and women, Belgium 2001-2004

**Dependent variables**:	Complaints of depression	
	**MEN**	**WOMEN**

	**B**	**B**

**Intercept**	-0.035 ***	0.005

**Contextual effects**		

**Unemployment rate**	0.046	0.112 ***
**Median area income (in euros)**	0.000	0.000
**Density**	0.194	-0.291

**Individual effects**		

**Social support**	-0.026 ***	-0.038 ***
**Current employment situation (ref.: paid labour)**		
*Retired*	0.019 ***	0.012 *
*Disabled*	0.109 ***	0.115 ***
*Unemployed*	0.024 ***	0.029 ***
*Househusband/wife*	-0.008	-0.011 *
*Student and others*	0.300 **	0.012
**Educational level (ref.: tertiary education)**		
*No diploma or primary education*	0.007	0.023 ***
*Lower secondary*	0.003	0.003
*Higher secondary*	-0.002	-0.001
**Equivalent income of the household (ref.: > 2.500 *€*)**		
*< 750 €*	0.016 *	0.028 ***
*750 - 1.000 €*	0.007	0.016 **
*1.000 - 1.500 €*	-0.001	0.011 *
*1.500 - 2.500 €*	-0.003	0.002
*Missing income*	-0.008	-0.012 *
**Age**	0.004 ***	0.004 **
**Household type (ref.: couple with child(ren))**		
*Single*	0.019 ***	0.007
*Single with child(ren)*	0.007	0.020 ***
*Couple*	0.004	-0.001
*Complex household*	0.008	0.000

*: p < .05; **: p < .01 ; ***: p < .001

**Table 3:** Multilevel regression analyses: association between residential area characteristics and complaints of generalised anxiety among men and women, Belgium 2001-2004

**Dependent variables**:	Complaints of generalised anxiety	
	**MEN**	**WOMEN**

	**B**	**B**

**Intercept**	-0.027 ***	-0.004

**Contextual effects**		

**Unemployment rate**	0.032	0.075
**Median area income (in euros)**	0.245	1.214
**Density**	-0.081	0.032

**Individual effects**		

**Social support**	-0.018 ***	-0.028 ***
**Current employment situation (ref.: paid labour)**		
*Retired*	0.003	0.003
*Disabled*	0.091 ***	0.101 ***
*Unemployed*	0.022 ***	0.022 ***
*Househusband/wife*	-0.028	-0.011 *
*Student and others*	0.032 **	0.102
**Educational level (ref.: tertiary education)**		
*No diploma or primary education*	0.008 *	0.038 ***
*Lower secondary*	0.009 *	0.017 ***
*Higher secondary*	-0.001	0.010 ***
**Equivalent income of the household (ref.: > 2.500 *€*)**		
*< 750 €*	0.018 **	0.017 *
*750 - 1.000 €*	0.009	0.013 *
*1.000 - 1.500 €*	0.002	0.005
*1.500 - 2.500 €*	-0.002	-0.002
*Missing income*	-0.008	-0.011 *
**Age**	-0.002	-0.002
**Household type (ref.: couple with child(ren))**		
*Single*	-0.001	-0.001
*Single with child(ren)*	-0.010	0.008
*Couple*	0.006	0.009 *
*Complex household*	0.004	0.003

*: p < .05; **: p < .01; ***: p < .001

Table 2 presents the results with regard to complaints of depression. At the residential area level, the local unemployment rate is related to complaints of depression and this relationship is only found among women *(B = 0.112, p < 0.001)*. On the individual level, it seems that male and female respondents who receive more social support report significantly less complaints of depression. This association is more pronounced among women *(men: B = -0.026, p < 0.001; women: B = -0.038, p < 0.001)*. As concerns the current employment status, it appears that the individual employment status is significantly associated with mental health complaints among both men and women. Men who are retired *(B = 0.019, p < 0.001)*, disabled *(B = 0.109, p < 0.001)*, unemployed *(B = 0.024, p < 0.001*) or student *(B = 0.300, p < 0.01) *all seem to have more complaints of depression, compared to the working population. Among women, the same phenomenon occurs with some small differences *(retired: B = 0.012, p < 0.05; disabled: B = 0.115, p < 0.001; unemployed: B = 0.029, p < 0.001*). The students do not significantly differ from the ones in paid labor, but apparently, housewives report less complaints of depression in comparison with the working population *(B = -0.011, p < 0.05)*. When we have a look at the impact of the level of education, we do not notice any significant associations among the male population. On the contrary, women who completed merely primary education report more complaints of depression (*B = 0.023, p < 0.001) *than women who attended tertiary education. With respect to household income, men of whom the household income is less than 750 euro a month report more complaints of depression *(B = 0.016, p < 0.05)*. Amongst women, more significant associations arise. Women with a household income lower than 1500 euro a month report more complaints of depression *(<750 €: B = 0.028, p < 0.001; 750-1000 €: B = 0.016, p < 0.01; 1000-1500 €: B = 0.011, p < 0.05) *compared to people in households who earn more than 2500 euro. In addition, our results show that older people report slightly more complaints of depression (men: *B = 0.004, p < 0.001; women: B = 0.004, p < 0.01*). Finally, being single is positively associated with the amount of complaints of depression reported among men *(B = 0.019, p < 0.001)*, compared to men who have a partner and children. Amongst women, singles with children report more complaints of depression compared to couples with children *(B = 0.020, p < 0.01)*.

Table 3 shows that neither one of the residential area characteristics is significantly associated with complaints of generalized anxiety. On the individual level, respondents who receive more social support report significantly less complaints of generalized anxiety both among men and women. Again the relationship is slightly more pronounced among the female population *(men: B = -0.018, p < 0.001; women: B = -0.028, p < 0.001)*. With regard to employment status, the same pattern arises as was the case with complaints of depression, except for people who are retired. They do not differ significantly from the working population. People who are disabled *(men: B = 0.091, p < 0.001; women: B = 0.101, p < 0.001)*, people who are unemployed *(men and women: B = 0.022, p < 0.001) *and male students *(B = 0.032, p < 0.01) *report more complaints of generalized anxiety than people in paid labor, while housewives report less complaints of generalized anxiety than the ones in paid labor *(B = -0.011, p < 0.05)*. People who completed merely primary education *(men: B = 0.008, p < 0.05; women: B = 0.038, p < 0.001) *and people who completed lower secondary education (*men: B = 0.009, p < 0.05; women: B = 0.017, p < 0.001) *report more complaints of generalized anxiety than people who attained tertiary education. However, the association is less clear-cut among the male population. Furthermore, women who attended higher secondary education also report more complaints of generalized anxiety in comparison with the ones who completed tertiary education *(B = 0.010, p < 0.001)*. With respect to income, a household income below 750 euro is related to more mental health complaints *(men: B = 0.018, p < 0.01; women: B = 0.017, p < 0.05)*, in comparison with people who earn more than 2500 euro. Women who have a household income between 750 and 1000 euro seem to suffer more from complaints of generalized anxiety than the highest income category *(B = 0.013, p < 0.05)*. As concerns household type, there only appears one significant difference in the female population. If women have a partner, but no children, they will be more likely to report more complaints of generalized anxiety than the ones who have a partner and children *(B = 0.009, p < 0.05)*.

## Discussion

This study explores the association between socio-economic characteristics of the residential area and complaints of depression and generalized anxiety, net of individual characteristics. The multilevel model was estimated for men and women separately, as gender differences are the focus of attention. The general Belgian population is studied, using individual-level data from the pooled Health Interview Surveys of 2001 and 2004, complemented with area-level data from Statistics Belgium and the General Socio-economic Survey. The data of both levels are merged by means of municipality codes.

Before we discuss the main findings, several limitations of this study need to be considered. A first limitation concerns the delineation of the geographical unit. In this study, the outline of the residential area is based on municipality codes. They were used because of the availability of the information, the sufficient amount of units and the diversity of the area-level characteristics. Furthermore, because not all municipalities were included in the sample, the threat of spatial autocorrelation was partially solved. Although census units are imperfect operationalizations of immediate living environments [77], they come closer than any commonly available spatial entity to represent the living environment. Secondly, despite the use of multilevel regression techniques, it remains difficult to distinguish between contextual and compositional influences. The environment operates through and interacts with individual characteristics. As a consequence, contextual effects are underestimated, as individual characteristics partly explain environmental influences [78,79]. The rather small value of the intra-class correlation of this study illustrates this phenomenon. However, this finding is consistent with other research that revealed that area-level characteristics merely explain a limited part of the variation in mental health outcomes [15,29,31]. Third, due to the cross-sectional design, this study is unable to confirm any causative statements, because it cannot rule out the selection hypothesis, which suggests that people with lower mental wellbeing may be drawn to live in less resourceful environments [49,80-82]. A fourth limitation is the fact that the impact of stress is not limited to any particular disorder, but can be expressed in differential ways. Women are more inclined to express distress by means of internalizing disorders, while men are more likely to react through antisocial and drinking behavior [83]. However, the differential expression hypothesis is tested in a related study, based on the same Belgian sample [50]. The results of this study revealed that living in a residential area with a high unemployment rate is more detrimental for women in terms of depression, but has the same impact on men and women when alcoholism is the mental health outcome.

Despite these shortcomings, several strengths can be emphasized. First, research concerning the association between contextual factors and mental health outcomes is rare among the general Belgian population. Second, the multilevel research design is quite unique, thanks to the clustering of the individual-level data, provided by the Health Interview Surveys and the area-level data obtained from Statistics Belgium and the General Socio-economic Survey. Third, studying the general population has the advantage that there exists greater variation regarding the amount of mental health complaints, compared to a clinical population. A less wide range of mental health complaints would make it more difficult to grasp subtle area-level associations [84].

The main findings that are obtained from this study are fourfold. First, the intra class correlation coefficient indicates that the residential area has a limited impact on the mental health of its inhabitants. Nevertheless, this finding is in line with previous research [3,4,25,26,83,84]. Most of the area-level variables accounted for only 1 to 5 percent of the variance in mental health outcomes. Second, the results at the individual level are in accordance with other studies, which gives an indication of the quality of the data [32,54,58,85-87]. Third, the multilevel regression analyses confirm that the local unemployment rate is associated with the amount of complaints of depression reported by its inhabitants, controlled for the individual employment status and the level of deprivation of the area. This result is in accordance with other empirical research [3,4,9,11,12]. The non-significance of the relationship between the median area income of the residential area and the mental health outcomes is congruent with the finding of Greiner et al. [62]. Fourth, a gender-specific pattern arises. The association between the local unemployment rate and complaints of depression is only significant among women. Women seem to be more sensitive of environmental strain, compared to men facing the same stressors. This finding confirms the study results of Day and Livingstone [88]. To explain this finding, we refer to the hypotheses of the social stress model. One possible explanation might be that women are more embedded in their local community due to the social roles they fulfill [9,42,43,89,90]. As a consequence, they are more exposed and vulnerable to events occurring in their social network. Kessler and McLead [91] define this as the high cost of caring hypothesis. Gilligan [92] builds on the theory of Chodorow and underlines that the feminine identity is characterized by solidarity or altruism, while the male identity is seen in terms of individualization. In addition, we have a look at the impact of social support, since it has been suggested by Dohrenwend and Dohrenwend [93] that women and men have different coping strategies to deal with stressful circumstances. This empirical model also underlines the relevance of available social support in the stress process. A multitude of empirical studies found negative relationships between social support and mental health problems. The negative relationship between social support and complaints of depression is found among both genders, which is consistent with other research [32,94,95]. This relationship is somehow more pronounced among the female population, but the available social support does not completely buffer the impact of the local unemployment rate on complaints of depression among women.

Finally, some implications for public health should be mentioned. Although the explained variance was limited, it should be kept in mind that characteristics of the residential area have a wide scope by affecting a large number of people. They complement the compositional effect of the individual characteristics of people living in the same residential area. Policy interventions that promote mental wellbeing and prevent mental illness should approach the male and female population differently and should address area-level features, especially in times of economic uncertainty. For example, the closure of a factory does not only have an impact on the mental wellbeing of the ones who were dismissed, but on the whole neighborhood.

## Competing interests

The authors declare that they have no competing interests.

## Authors' contributions

EP responded to the comments of the referees, did a rerun of the analyses and rewrote the article to its present form. LVP performed the statistical analysis and drafted the original version of the manuscript. MV and KL commented on the statistical design and on the structure of the text. PB coordinated the project and helped with the design of the study. All authors have read and approved the final manuscript.
